# HHV-8 Seroprevalence and Genotype Distribution in Africa, 1998–2017: A Systematic Review

**DOI:** 10.3390/v10090458

**Published:** 2018-08-27

**Authors:** Elizabeth M. Etta, Doyinmola P. Alayande, Lufuno G. Mavhandu-Ramarumo, George Gachara, Pascal O. Bessong

**Affiliations:** 1 HIV/AIDS & Global Health Research Programme, University of Venda, Thohoyandou 0950, South Africa; queenettamashu@gmail.com (E.M.E.); doyin_alayande@yahoo.com (D.P.A.); lufuno.mavhandu@univen.ac.za (L.G.M.-R.); 2 Department of Medical Laboratory Sciences, Kenyatta University, Nairobi 34556-00100, Kenya; ggachara@gmail.com

**Keywords:** HHV-8, seroprevalence, genotypes, systematic review, Africa

## Abstract

Human herpes virus type 8 (HHV-8) is the causative agent of Kaposi’s sarcoma (KS). We systematically reviewed literature published between 1998 and 2017, according to the PRISMA guidelines, to understand the distribution of HHV-8 infection in Africa. More than two-thirds (64%) of studies reported on seroprevalence and 29.3% on genotypes; 9.5% were on both seroprevalence and genotypes. About 45% of African countries had data on HHV-8 seroprevalence exclusively, and more than half (53%) had data on either seroprevalence or genotypes. Almost half (47%) of the countries had no data on HHV-8 infection. There was high heterogeneity in the types of tests and interpretation algorithms used in determining HHV-8 seropositivity across the different studies. Generally, seroprevalence ranged from 2.0% in a group of young children in Eritrea to 100% in a small group of individuals with KS in Central African Republic, and in a larger group of individuals with KS in Morocco. Approximately 16% of studies reported on children. Difference in seroprevalence across the African regions was not significant (95% CI, χ^2^ = 0.86; *p* = 0.35), although specifically a relatively significant level of infection was observed in HIV-infected children. About 38% of the countries had data on K1 genotypes. K1 genotypes A, A5, B, C, F and Z occurred at frequencies of 5.3%, 26.3%, 42.1%, 18.4%, 5.3% and 2.6%, respectively. Twenty-three percent of the countries had data for K15 genotypes, and genotypes P, M and N occurred at frequencies of 52.2%, 39.1%, and 8.7%, respectively. Data on HHV-8 inter-genotype recombinants in Africa are scanty. HHV-8 may be endemic in the entire Africa continent but there is need for a harmonized testing protocol for a better understanding of HHV-8 seropositivity. K1 genotypes A5 and B, and K15 genotypes P and M, from Africa, should be considered in vaccine design efforts.

## 1. Introduction

Human herpes virus type 8 (HHV-8) is the causative agent of four classes of Kaposi’s sarcoma (KS) [1]: endemic, classic, iatrogenic and AIDS-associated KS; of these, endemic-KS and AIDS-KS are the most aggressive. AIDS-KS has been highlighted in young homosexual men [2], while classic-KS is common in elderly Mediterranean people and individuals of Eastern Europe [3]. Furthermore [4], reported on endemic-KS, also called African endemic-KS, as common in children and young adults in sub-Saharan Africa. Iatrogenic-KS has been observed in immunosuppressed patients who had undergone a solid organ transplant [5,6].

Globally, the epidemiologic pattern of HHV-8 is uneven, but follows that of KS, and countries at high risk of KS report high prevalence of HHV-8. In a healthy population, there is great variability in the seroprevalence of HHV-8, as opposed to groups at increased risk of developing KS. HHV-8 undergoes both latent and lytic phases in its life cycle, whereby the virus remains within the host at a dormant stage until other cofactors trigger it to start replicating, leading to the lytic phase [7,8,9]. The mode of transmission of HHV-8 can either be vertical or horizontal, including mother to child transmission and from members of family units [10,11,12].

HHV-8 infection is ubiquitous in African regions with increased incidences of endemic Kaposi’s sarcoma in the general population and AIDS-associated Kaposi’s sarcoma in the HIV/AIDS population [13,14]. An increase in the incidence of KS has been observed due to HIV infection in Africa. However, with the scale up and improved access to combination antiretroviral therapy, it is expected that the incidence of KS may be mitigated as has been observed in the developed world [15,16]. In contrast to observations in US, studies have showed that HHV-8 seroprevalence increases from childhood to adulthood in African regions [17,18,19]. Treatment for HHV-8 or KS includes combined chemotherapy, such as vincristine, bleomycin and doxorubicin, radiation therapy, surgery and biological therapy that will effectively dissolve lesions whether localized or widespread, compared to a single HAART drug [20,21,22].

HHV-8 is characterized by high genetic variability across the entire genome, with the highest level of genetic variation observed at the 5′ and 3′ ends of the genome. HHV-8 has been classified into genotypes A, A5, B, C, D, E, F and Z based on the hypervariable regions (VR1 and VR2) of the *K1* gene; while genotypes P, M and N are based on the *K15* gene. Generally, these genotypes have been identified globally [4,23,24,25,26,27,28,29,30,31]. Genotypes B, Q, R and N are ORF26 genotypes, of which genotypes B and N overlap with genotypes based on *K1* and *K15* genes.

There are several ongoing vaccines and therapeutic development efforts against HHV-8 [32,33,34]. In this backdrop, it is important to understand the burden of HHV-8 in Africa where the infection appears to be relatively common. The current systematic review examined and analyzed data on the prevalence and molecular epidemiology of HHV-8 in all African countries from 1998–2017.

## 2. Methodology

### 2.1. Inclusion Criteria for Study Analysis

We conducted a systematic review of published full text articles on HHV-8 seroprevalence and genotypes from 53 African countries, according to the PRISMA guidelines, except for meta-analysis. Sudan and South Sudan were considered as one country. Cross-sectional, case report, retrospective, prospective and observational studies on HHV-8 seroprevalence and/or genotypes were included for analysis. Full texts in the French language were interpreted, analyzed and included in the analysis. However, there were no full text articles in other languages that met the inclusion criteria. African countries were assessed and categorized into Central, East, North, Southern and West Africa.

### 2.2. Relevant Literature Searches

MEDLINE, EMBASE, SCOPUS, WEB OF SCIENCE databases and conference proceedings, were searched for published data from 1998–2017. Using electronic search, reference lists were screened for relevant and additional data.

### 2.3. MEDLINE Search Strategy Using PubMed

The search terms used were: ((((((prevalence* OR epidemiology* OR incidence OR seroprevalence* OR seroepidemiology* OR sero-epidemiology* OR seropositivity* OR sero-positivity*)) OR ((((sero-epidemiologic studies [MeSH Terms] OR prevalence [MeSH Terms] OR incidence [MeSH Terms]))))) AND ((((((((herpesvirus 8, human [Term]) OR “human herpesrvirus 8”) OR “HHV8”) OR “HHV-8” OR “KSHV” OR “kaposi sarcoma associated herpesvirus”) OR “kaposi’s sarcoma associated herpes-virus”) OR “kaposi sarcoma-associated herpesvirus”) OR “kaposi’s sarcoma-associated herpesvirus”) OR “kaposi sar-coma herpesvirus”) OR kaposi virus))))) NOT ((animals[mh] NOT humans[mh])). In addition, each search strategy was complemented with other search criteria as follows: reviews of HHV-8/KSHV OR HHV-8 review and epidemiology OR genetic characterization of HHV-8/KSHV, genetic OR characterization of KSHV and genotype distribution of HHV-8 K1/K15. All these search options were accompanied with an African country (name). This was repeatedly done for all 53 African countries.

### 2.4. EMBASE Search Strategy Using Science Direct

The search terms were: #1.1 “human herpesvirus 8”/exp OR “human herpesvirus 8” OR “HHV-8” OR “HHV8” OR “KSHV” OR “kaposi sarcoma associated herpesvirus”/exp OR “kaposi sarcoma associated herpesvirus” OR “kaposi sarcoma-associated herpesvirus”/exp OR “kaposi sarcoma-associated herpesvirus” OR kaposi NEXT/3 herpesvirus OR kaposi NEXT/3 virus AND [embase]/lim #1.2 “prevalence”/exp OR prevalence OR “seroprevalence”/exp OR seroprevalence OR “incidence”/exp OR incidence OR “seroepidemiology”/exp OR seroepidemiology AND [embase]/lim #1.3 prevalen* OR inciden* OR epidemiolog* OR sero*epidemiolog* OR sero*prevalen* OR sero*positiv* AND [embase]/lim #1.4 #1.2 OR #1.3 #1.5 #1.1 AND #1.4 #1.6 “animals”/exp NOT “humans”/exp #1.7 #1.5 NOT #1.6.])). In addition, each search strategy was concluded with additional options as thus: reviews of HHV-8/KSHV OR HHV-8 review and epidemiology OR genetic characterization of HHV-8/KSHV, genetic OR characterization of KSHV and genotype distribution of HHV-8 K1/K15. All these search options were accompanied with an African country (name). This was repeatedly done for all 53 African countries.

### 2.5. SCOPUS Search Strategy Using Elsevier

Search strategies were as follows: seroprevalence OR seroepidemiology OR sero-epidemiology OR seropositivity OR seropositivity OR sero-epidemiologic studies OR prevalence OR incidence AND herpesvirus 8, human OR human herpesrvirus 8 OR hhv-8 OR hhv-8 OR kshv OR kaposi sarcoma associated herpesvirus OR kaposi’s sarcoma associated herpes-virus OR kaposi sarcoma-associated herpesvirus OR kaposi’s sarcoma-associated herpesvirus OR kaposi sarcoma herpesvirus/kaposi virus OR reviews of HHV-8/KSHV OR HHV-8 review and epidemiology OR genetic characterization of HHV-8/KSHV OR genetic characterization of KSHV and genotype distribution of HHV-8 K1/K15. All these search options were accompanied with an African country (name). This was repeatedly done for all 53 African countries.

### 2.6. Web of Science Search Strategy Using Analytic Database

Search strategies were as follows: seroprevalence OR seroepidemiology OR sero-epidemiology OR seropositivity OR sero-positivity OR seroepidemiologic studies OR prevalence OR incidence and herpesvirus 8, OR human herpesvirus 8 OR HHV-8 OR KSHV OR kaposi sarcoma associated herpesvirus OR kaposi’s sarcoma associated herpesvirus OR kaposi associated herpes virus OR kaposi’s sarcoma associated herpes virus OR kaposi sarcoma herpesvirus OR kaposi sarcoma virus, reviews on epidemiology of HHV-8/KSHV, genetic characterization of HHV-8/KSHV genotype distribution of HHV-8 K1/K15. All these search options were accompanied with an African country (name). This was repeatedly done for all 53 African countries.

### 2.7. Searching Other Sources

Additional searches were for additional data and the focus was on African countries. We screened reference lists of relevant papers and searched the following conferences: Annual Meeting of the American Society of Clinical Oncology (ASCO) (http://www.asco.org/ASCOv2/ Meetings); International Conference on Malignancies in AIDS and Other Acquired Immuno-deficiencies (http://www.capconcorp.com/meeting/2013/14thICMAOI/index.asp); Conference on Retroviruses and Opportunistic Infections (CROI) (www.croi2017.org).

### 2.8. Data Extraction

**Figure 1:**
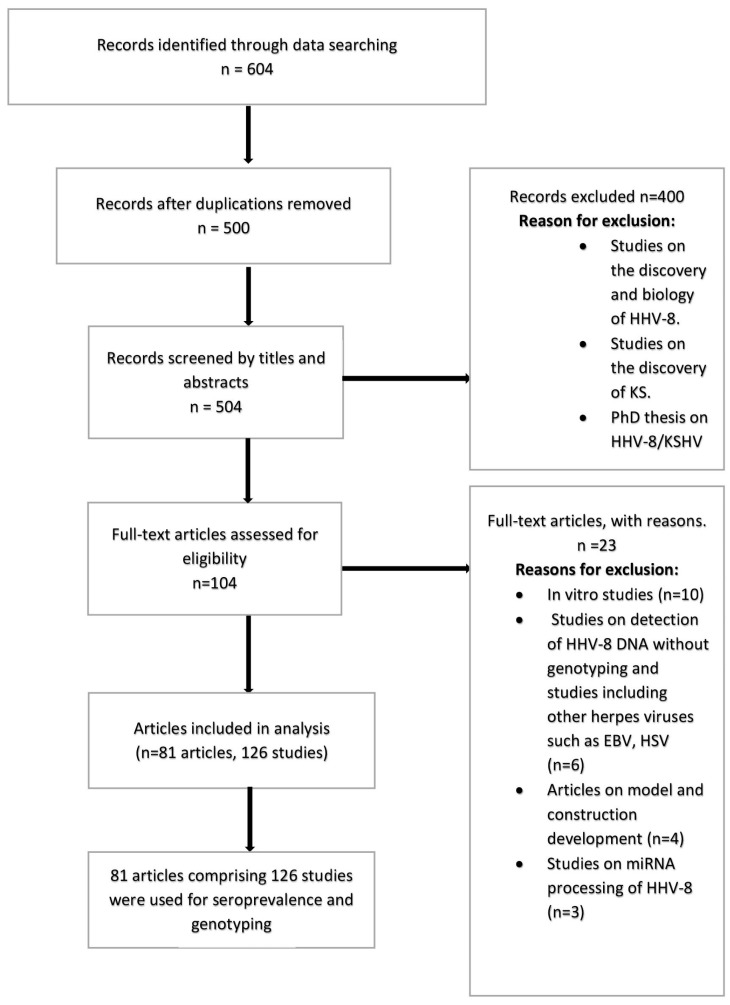
Flow chart on the selection of studies included for analysis.

Two reviewers downloaded relevant articles from the above search options and classified each article using an article grid. These classifications were based on: the study objectives, design, sampling size, results, discussion and conclusion. For all included studies, information was extracted on: author(s), study type, country of study, country of participants, study population, sample size, sample type, number of males, number of females, number of children, type of assay, seroprevalence, source of DNA, K1 genotypes and K15 genotypes. We used ‘X’ for parameters with no information. The various tests used for the determination of HHV-8 seropositivity were also considered. These tests were immunofluorescence (IFA) and enzyme immunoassay (EIA), immuno-peroxidase (IMP), western blots and antigen ELISA tests (latent ORF73 and lytic K8.1). In addition, relevant articles on the genetic characterization of HHV-8 for *K1* and *K15* genes were analyzed. Figure 1 shows a PRISMA flow chart on the selection of studies included for analysis.

## 3. Results

The use of different approaches and algorithms to determine the sero-epidemiology of HHV-8 across different populations presents challenges for comparisons. Examples of these challenges include the use of different dilution factors in plasma or serum preparation for antibody detection, the use of ELISA with different principles and interpretation guidelines, different immunofluorescence techniques, or a combination of these approaches. In addition to these, the inclusion of different populations (low and high risk), even in the same study community, increases the degree of complexity in deciphering an accurate picture of the distribution of HHV-8 infection among populations. Consequently, herein, we firstly present a descriptive analysis of the systematic literature search on studies on HHV-8 sero-epidemiology in Africa. To extract salient sero-epidemiological features, amid the challenges posed by different methodologies, we performed, where plausible, comparable analysis for studies involving children, pregnant women, and for rural and urban populations, against the backdrop of the risk factors for HHV-8 acquisition, for the different regions of Africa (Southern, Central, West, East, and North Africa). Countries were categorized as follows: Southern Africa—Botswana, Zambia, Mozambique, South Africa, Zimbabwe, Namibia, Swaziland, Lesotho, Malawi; Central Africa—Angola, Cameroon, Central Africa Republic, Chad, Congo, Gabon, Democratic Republic of Congo, Equatorial Guinea, Sao Tome & Principle; West Africa—Nigeria, Burkina Faso, Guinea, Gambia, Ghana, Mali, Senegal, Sierra Leonne, Niger, Liberia, Benin, Cote d’Ivoire, Togo, Cape Verde, Mauritania; East Africa—Tanzania, Kenya, Uganda, Ethiopia, Rwanda, Burundi, Djibouti, Eritrea, Somalia, Madagascar, Comoros; and, North Africa—Algeria, Egypt, Tunisia, Sudan (including South Sudan), Morocco, Libya, Western Sahara.

### 3.1. Characteristics of Studies Included in the Analysis

We identified a total of 604 articles from database searches, which were reduced to 500 after the removal of duplicates. Observational studies included for analysis were cross-sectional, prospective, retrospective and case report, which occurred at different settings such as rural, urban or hospital based. Out of the 500 articles, 81 articles met the inclusion criteria and were further analyzed (Figure 1). Some studies reported on multi-country and multi-site studies. One hundred and twenty-six studies on HHV-8 sero-epidemiology or genotypes, published from 1998–2017, were obtained from the 81 articles and met the inclusion criteria for analysis. About 64% (81/126) of the studies were available on HHV-8 seroprevalence; 29.3% (37/126) were available on HHV-8 genotypes; and 9.5% (12/126) of the studies were available for both seroprevalence and genotypes. Overall, 52.8% (28/53) of African countries had data on either seroprevalence or genotypes of HHV-8. Tables S1–S5 (Supplementary Materials) present studies from the different African regions included in the analysis.

### 3.2. HHV-8 Seroprevalence Distribution in Africa

**Figure 2:**
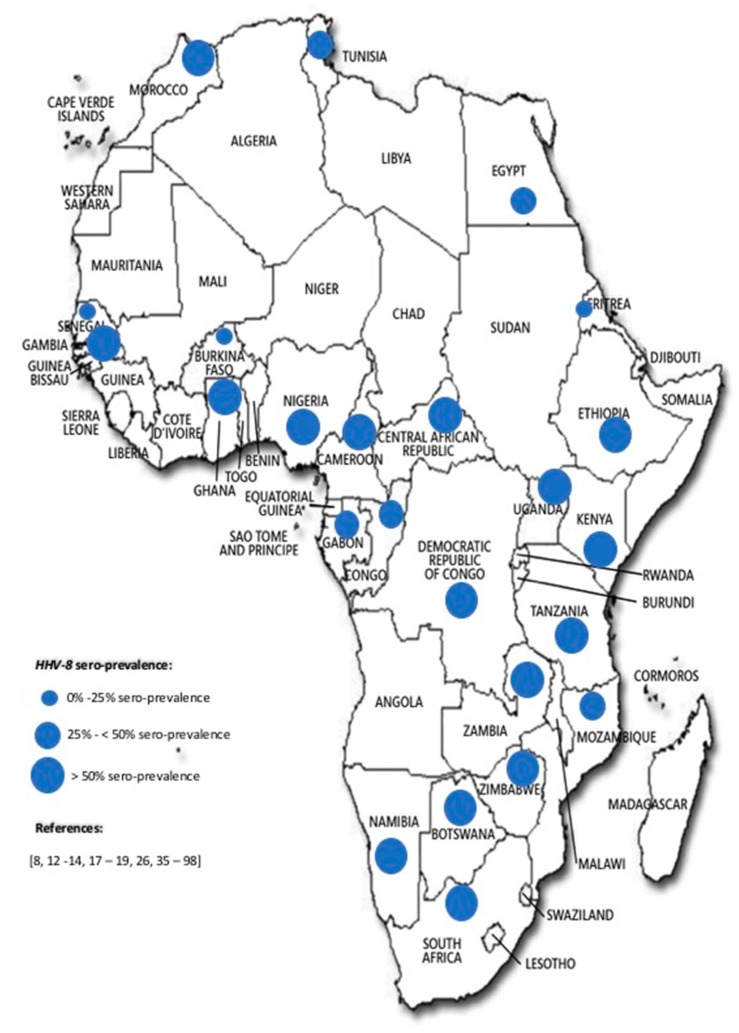
A representation of the highest HHV-8 seroprevalence observed in any one population in African countries (1998–2017). It should be borne in mind that the reported seroprevalences are from studied populations which employed different designs, approaches, populations, tests and interpretation algorithms, even within the same country. Thus, it could be difficult to make direct comparisons between and among studied populations [8,12,13,14,17,18,19,26,35,36,37,38,39,40,41,42,43,44,45,46,47,48,49,50,51,52,53,54,55,56,57,58,59,60,61,62,63,64,65,66,67,68,69,70,71,72,73,74,75,76,77,78,79,80,81,82,83,84,85,86,87,88,89,90,91,92,93,94,95,96,97,98].

About 45% (24/53) of African countries had data on HHV-8 seroprevalence only. Bearing in mind that different test strategies were applied in determining seropositivity in different populations, seropositivity reported ranged from 0.0% in a group of blood donors in Morocco by indirect immunofluorescence [35], through 2.0% in a group of young children in Eritrea by the detection of antibodies to ORF73 [36], to 100% in a small group of individuals with KS in the Central Africa Republic using immunofluorescence and immuno-peroxidase assays [37], although in a larger group of individuals with KS in Morocco the prevalence was 92% by indirect immunofluorescence [35]. Out of the 126 studies, 20 (15.8%) reported on children, in which seroprevalence ranged from 2% to 69%. Regionally, the seroprevalence across the continent ranged as follows: Southern Africa (14.0–90.0%), Central Africa (17.4–100.0%), West Africa (14.0–83.1%), East Africa (2.0–93.0%), and North Africa (0.0–92.0%). Considering the highest reported seroprevalence from the different African regions, there was no significant difference among the African regions (95% CI, χ^2^ = 0.86; *p* = 0.35). Figure 2 represents the relative seroprevalence of HHV-8 across Africa.

### 3.3. A Comparison of HHV-8 Seroprevalence among Selected Populations across African Regions

From the available data, HHV-8 infection among children was observed to be significantly different across the African regions, with children in Central Africa being the most infected. The burden of infection was also significant in HIV-infected children compared to HIV non-infected children in Southern Africa (Table 1). Across the different African regions, HHV-8 antibodies were generally detected in a higher frequency in non-pregnant women than in pregnant women of comparable age, except in West Africa, were pregnant women were more infected than non-pregnant women (Table 2). No studies meeting the inclusion criteria were available for North Africa. Regarding geographical setting, there is no clear picture on the distribution of HHV-8 between rural and urban populations within and across African regions. For example, the urban population was marginally more infected than the rural population in Southern and East Africa, while this is not the case in West Africa. Across regions, the urban population in West Africa was the most infected. Data for North and Central Africa were inadequate to permit meaningful comparisons (Table 3).

### 3.4. HHV-8 Genotype Distribution in Africa

**Figure 3:**
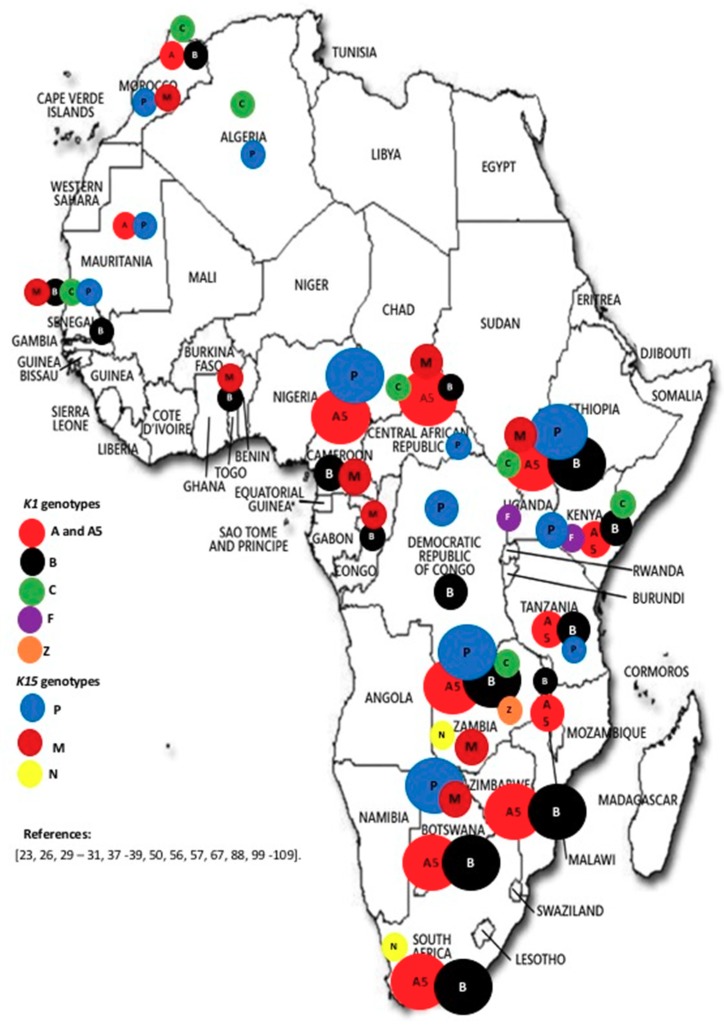
Map of Africa showing the occurrence of HHV-8 genotypes. There appears to be a general distribution of different genotypes across the continent. Of note is the identification of K15 genotype N in Southern Africa only (Zambia and South Africa). Countries without indications had no published data on HHV-8 genotypes between 1998–2017. The size of the circles indicates the relative proportion of occurrence of the different genotypes in the different countries. Overall, K1 genotypes A5 and B, and K15 genotypes P and M are the most common throughout the continent [23,26,29,30,31,37,38,39,42,44,50,56,57,67,88,99,100,101,102,103,104,105,106,107,108,109].

**Figure 4:**
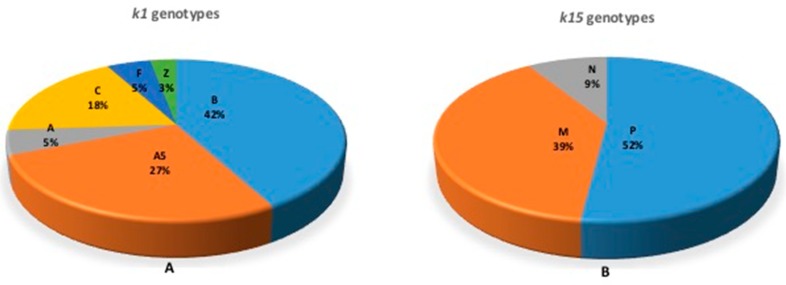
Proportional representation of HHV-8 genotypes in Africa. (**A**) represents K1 genotypes; (**B**) represents K15 genotypes. K1 genotype B is the most prevalent followed by genotype A5, while K15 genotype P is most prevalent genotype followed by genotype M.

Based on the *K1* gene, 33.9% (18/53) of African countries had data on genotypes, while 28.3% (15/53) of countries had data on K15 genotypes (Figure 3). Genotypes A, A5, B, C, F and Z were identified at frequencies of 5.3% (2/38), 26.3% (10/38), 42.1% (16/38), 18.4% (7/38), 5.3% (2/38) and 2.6% (1/38), respectively; genotypes P, M and N of the *K15* gene were identified at frequencies of 52.2% (12/23), 39.1% (9/23) and 8.7% (2/23), respectively (Figure 4). Of the 18 countries from which K1 genotypes were reported, at least two genotypes were reported in each. Genotype Z was reported from only one study in Zambia involving children [23]. All other genotypes were reported in both children and adults. From the available data, there appears to be no trend in the distribution of genotypes across the continent. Worthy of note though is the description of K15 genotype N in Southern Africa (South Africa and Zambia) [23,99], and it is not clear whether K15 genotype N is restricted to Southern Africa. Data were reported for a few countries on intra-genotypic variants but data on inter-genotypic recombinants appears to be scanty [38,39,100,101].

## 4. Discussion and Conclusions

Studies on HHV-8 infection are important in regions, such as Africa, where a significant proportion of the continent is infected with immunosuppressive agents, such as HIV. This review was aimed at providing an update on the epidemiology and prevalent genotypes of HHV-8 in Africa. We observed that infection with HHV-8 is generally highly endemic in most parts of Africa, indicating that risk factors for infection persist throughout the continent. Reports suggest that there is variety and non-uniformity in the modes of transmission of HHV-8 among populations in Africa. For example, horizontal transmission from mother to child through chewed food (saliva) and contact with family members [12,42,89] sexual intercourse [62,65,87] other infections such as malaria [83], and blood transfusion [90,93] have been identified as risk factors for transmission. High seropositivity has been shown in pregnant women [64], and it has been suggested that the risk of HHV-8 seropositivity is significantly higher in children of HHV-8 seropositive mothers compared with children of HHV-8 seronegative mothers [110]. Further to this, in a sub-analysis of literature of pregnant and non-pregnant women, we found that in certain regions of Africa HHV-8 seropositivity was more frequent in non-pregnant women than in pregnant women of comparable age, while the converse was true in other regions (for example, in West Africa). This discrepancy or lack of trend may be attributed to other underlying confounding factors in HHV-8 acquisition in the different study populations and regions. Generally, there is a relatively lower rate of infection among children in Africa, which reflects findings that the rate of infection with HHV-8 in a given population increases in older age groups [71]. Most of the countries in Central, Southern and East African regions of the continent harbor huge burdens of HHV-8 infection. Geographic and cultural factors have been proposed as key determinants of HHV-8 transmission in some of these regions [12,53,54], also accounting for the variability in prevalence across and within geographic regions [71]. Additionally, a meta-analysis involving studies undertaken in 32 countries in sub-Saharan Africa, Australia, North and South America, Europe and Asia, demonstrated that HIV-infection is associated with a significant increase in HHV-8 co-infection globally, and in all population groups [111]. This scenario thereby coexists with the high HIV endemicity in Central, East and Southern Africa, and the probability of co-infection is therefore significantly increased in the said populations. Hence, with immunosuppression from HIV infection, it is plausible that cases of Kaposi’s sarcoma will rise, although this could be mitigated with the improved access to antiretroviral therapy [45].

Our analysis revealed that HHV-8 genotypes A5 and B based on the *K1* gene and genotypes P and M based on the *K15* gene are the most prevalent in Africa. Genotype B is associated with endemic KS, while genotype P has been reported to be highly transmissible. Previous reports [25,27,43], indicated the clustering patterns of HHV-8 genotypes with ethnic composition of the population and geographical location, and these might have arisen through ancient human migrations and genetic polymorphisms. The classification of HHV-8 into genotypes has not generally followed a unified approach. Some studies have used more than one gene for viral grouping, and some investigators has shown a link between gene regions; for example, [30] reported a link between ORF26 and K1. Intra-genotype variants have also been reported; for example, K1 A5 and K15 M based on allele differences [38,39,88]. A5 viruses are closely related to viruses found mostly in viral populations that form the A1-4 genotypes. Previously, A5 was thought to have emerged from recombination. However, subsequent and more robust analyses point to the emergence of the A5 genotype because of natural genetic drift and divergence between A1-4 genotypes [39,65]. It has been hypothesized [28] that the distribution of the K1-A5 genotype in African populations is because of a “very rapid and recent aggressive spread” of the K1-A5 prototype introduced into the population, aided by a selective advantage of the A5 allele. Overall, previous studies have demonstrated the clustering patterns of HHV-8 genotypes with geography and ethnicity, and these may have arisen through ancient human migrations and genetic polymorphisms, respectively [100,101]. The current analysis revealed scanty data on inter-genotype recombinants, with two studies reporting on the A/C genotype in Africa [39,101]. The A/C genotype is prevalent in Europe, United States, Asia, and the Middle East.

The current analysis, covering the period 1998–2017, suffers from a relative lack of data, dispersed across the continent, from about 50% of the countries, thereby somewhat limiting our appreciation of the burden of HHV-8 infection. However, the fact that high prevalence was noted in certain countries in all regions of the continent suggests that there is generalized infection across the geographical spectrum of Africa. The findings suggest that the entire continent is endemic for HHV-8, and co-infection with HIV may be common in those African countries endemic for HIV. Noting that there is high heterogeneity in the testing strategies employed in the detection of HHV-8 antibodies, there is need for an acceptable harmonized protocol to enhance the possibility of comparisons of seropositivity across studies. Since genotypes A5, B, P and M are highly prevalent in Africa, it is suggested that full length genomes of these genotypes from Africa be characterized to support the rational selection of genes for the design and development of vaccine candidates. This is, apparently, the first attempt at a systematic review of the seroprevalence and genotype distribution of HHV-8 comprising all African countries.

## Figures and Tables

**Table 1:** Relative HHV-8 infection burden in children, and proportion of HIV positive, HIV negative, and children with KS among the child population in Africa.

African Region	Percentage of Children Infected with HHV-8	Percentage of HIV Positive Children Infected with HHV-8 of All Children with HHV-8	Percentage of HIV Negative Children Infected with HHV-8 of All Children with HHV-8	Percentage of Children with KS and Infected with HHV-8 of All Children with HHV-8	Chi-Square Value	*p*-Value (Significant at *p* ≤ 0.05)
Southern	31.4 (342/1092)	52.6 (180/342)	21.1 (72/342)	26.3 (90/342)	90.5	< 0.0015
Central	49.5 (329/665)	No data	No data	No data	Not done	Not done
West	11.4 (49/429)	18.6 (80/429)	81.3 (349/429)	No data	128.8	0.0001
East	20.7 (945/4557)	No data	No data	1.6 (15/945)	Not done	Not done
North	43.3 (122/282)	No data	No data	5.3 (15/282)	Not done	Not done
Chi-square value	367.2	98.3	279.1	220.0	34.9	0.401
*p*-value	< 0.00001	-	-	-	-	-

Data on children described in selected studies were summed up for each region and analysed. The percentages of HIV positive children, HIV negative children, and children with KS are calculated based on the total number of children with HHV-8 pooled from the extracted studies in the different African regions. Data from North Africa were not sufficient for meaningful comparable analysis and were omitted.

**Table 2:** Relative HHV-8 infection burden in women of comparable age (25–45 years) in Africa.

African Region	Percentage of Women Infected with HHV-8	Percentage of Pregnant Women Infected with HHV-8 of All Women with HHV-8	Percentage of Non-Pregnant Women Infected with HHV-8 of All Women with HHV-8	Chi-Square value	*p*-Value (Significant at *p* ≤ 0.05)
Southern	28.7 (4196/14,612)	8.2 (343/4196)	91.8 (3853/4196)	155.93	<0.00001
Central	6.2 (287/4626)	27.5 (79/287)	72.5 (208/287)	100.00	<0.00001
West	1.7 (151/8491)	61.0 (92/151)	39.3 (59/151)	79.20	0.00001
East	26.1 (2280/8729)	22.2 (501/2280)	78.0 (1779/2280)	80.73	0.00001
Chi Square value	41.49	72.54	41.61	-	-
*p*-value	<0.00001	<0.0001	<0.00001	-	-

Data on women described in selected studies were summed up for each region and analysed. The percentages of pregnant and non-pregnant women infected with HHV-8 were calculated based on the total number of women with HHV-8 pooled from the extracted studies in the different African regions. Data from the North Africa region were not sufficient for meaningful comparable analysis and were omitted.

**Table 3:** Relative HHV-8 infection burden in rural and urban populations in Africa.

African Region	Percentage of HHV-8 Seropositivity in the Rural Setting	Percentage of HHV-8 Seropositivity in the Urban Setting	Chi-Square Value	*p*-Value (Significant at *p* ≤ 0.05)
Southern	5.4 (207/3781)	3.3 (191/5710)	764.03	0.00001
Central	20.0 (516/2579)	No data	Not done	Not done
West	31.8 (77/242)	42.0 (26/62)	2.4462	0.119
East	29.0 (829/2409)	42.5 (450/1060)	84.52	0.0
Chi Square value	52.30	50.21	Not done	Not done
*p*-value	<0.00001	<0.0001	Not done	Not done

Data on the rural and urban populations described in the selected studies were summed up for each region and analysed. Data from the North African region were not sufficient for meaningful comparable analysis and were omitted.

## Supplementary Materials

The following are available online at http://www.mdpi.com/1999-4915/10/9/458/s1. Tables S1–S5: Characteristics of studies included in the systematic review according to regions of Africa.
