# Supplementary material for: HHV-8 Seroprevalence and Genotype Distribution in Africa, 1998–2017: A Systematic Review
Source: Viruses. 2018 Aug 27;10(9):458. doi: 10.3390/v10090458 (PMC6164965; doi:10.3390/v10090458)
Supplement: Supplementary file 1 [file viruses-10-00458-s001.pdf]

Table S1: Characteristics of studies on Southern African countries included in the analysis

Reference	Study type	Country of study	Country of participants	Study population	Sample size	Type of assay for sero-prevalence study	No of males	No of females	No of children	Average Age(Age range of participants)	Percentage sero-prevalence	Source of DNA/Antibodies for serology/genotyping	K1 genotypes	K15 genotypes
[26]	Retrospective	Ecuador	Botswana	Rural based volunteer (Bantu)	160	K8.1 ELISA and LANA IFA	N/M	N/M	N/M	45 (15 -75 years)	ELISA=76% IFA =90%	Blood (sera & buffy coat)	A5, B	P
[26]	Retrospective	Ecuador	Botswana	Rural based volunteer (San)	156	K8.1 ELISA and LANA IFA	N/M	N/M	N/M	45 (15 -75 years)	ELISA=88% IFA =79%	Blood (sera & buffy coat)	B	P, M
[40]	Retrospective	Botswana	Botswana	HIV-AIDS hospital based patients	22	LANA ELISA	N/M	22	N/M	54 (29 -79 years)	61%	X	X	X
[41]	Retrospective	USA	Botswana	Community based (San)	155	ELISA and IFA	81	69	N/M	36.5 (18 -55 years)	87%	Serum/Plasma	X	X
[41]	Retrospective	USA	Botswana	Community based (Bantu)	161	ELISA and IFA	89	80	0	N/M	76%	Serum/Plasma	X	X
[23]	Prospective	Zambia	Zambia	FI &Endemic KS children, hospital based	200	X	N/M	N/M	200	20 (6 -34 months)	X	Biopsy & whole blood	A5, B, C, Z	P, M
[42]	Retrospective	UK	Zambia	HIV +ve, blood donors and KS patients	30<br>116	ELISA, IFA and WB	25<br>98	5<br>18	0<br>0	27 (18-54 years)<br>38 (16 -70 years)	74%	KS biopsy	A5, B, C	P, M
[43]	Prospective	Zambia	Zambia	Hospital based	1425	IFA	0	1425	N/M	23.5 (23 -24 years)	39.9%	Plasma	X	X
[44]	Retrospective	Zambia	Zambia	Hospital based	5	X	5	0	0	N/M	X	Biopsy	B	X
[102]	Retrospective	USA	Zambia	Hospital based patient	5	X	5	0	0	N/M	X	Biopsy	X	P

[45]	Retrospective	USA	Zambia	Hospital based patients	40	ELISA	22	18	0	42.5 (17 -68 years)	37.5%	Serum	X	X
[46]	Retrospective	USA	Zambia	Hospital based patients	251	IFA and WB	98	153	0	49 (14 -84 years)	58%	X	X	X
[47]	Cross sectional study	USA	Zambia	Population based	378	IFA	0	378	N/M	30 (15 -45 years)	48.4%	X	X	X
[23]	Prospective	Zambia	Zambia	HIV +ve children	287	X	N/M	N/M	287	30 (15 -45 years)	X	Biopsy & blood	A5, B	P, N
[38]	Prospective	Brazil	Zambia	Cross sectional survey	788	ELISA	N/M	N/M	N/M	23.5 (2 -45 years)	14%	Plasma & Saliva	A5, B, C	X
[48]	Retrospective	Mozambique	Mozambique	Hospital based	136	ELISA	58	78	0	36.5 (28 -45 years)	44.7%	X	X	X
[49]	Prospective	Mozambique	Mozambique	General population	752	ELISA	263	489	0	30 (10 -50 years)	21.4%	X	X	X
[14]	Retrospective	England	South Africa	Hospital based cancer patients	3293	IFA	N/M	N/M	N/M	39 (15 -65 years)	32%	Serum	X	X
[19]	Retrospective	South Africa	South Africa	Hospital based	136	ELISA, IFA and WB	50	50	36	9.5 (1 -18 months)	38-95%	Serum	X	X
[31]	Retrospective	South Africa	South Africa	AIDS-KS + Endemic KS patients	86	X	53	33	0	36.5 (31 -42 years)	X	KS biopsy	A1, A5, B1, B2, B3	X
[50]	Prospective	South Africa	South Africa	Cross sectional HIV + naïve children	416	ELISA	0	0	416	5.2 (1.5 – 8.9 years)	28.5%	Plasma	X	X
[51]	Prospective	South Africa	South Africa	Population based	427	ELISA	0	427	0	37 (31 – 43 years)	7.5 - 9%	Plasma	X	X
[52]	Prospective	South Africa	South Africa	Hospital based	N/M	ELISA	N/M	N/M	N/M	N/M	48%	Plasma	X	X
[53]	Retrospective	South Africa	South Africa	Pregnant women, hospital based	1740	ELISA	0	1740	0	39.5 (16 -63 years)	47.5%	X	X	X

[54]	Retrospective	USA	South Africa	Pregnant women, with KS	1228	ELISA	0	1228	0	39.5 (16 -63 years)	36.1%	X	X	X
[54]	Retrospective	USA	South Africa	Pregnant women, with KS	1228	ELISA	0	1228	0	36.5 (24 – 45) years)	19.9%	X	X	X
[99]	Retrospective	South Africa	South Africa	AIDS-KS; hospital based	40	X	30	10	0	41.5 (25 -58 years)	X	Biopsy	A5, B1	N*
[55]	Retrospective	South Africa	South Africa	Survey	2191	ELISA	0	2191	0	N/M	39.1%	Serum	X	X
[56]	Retrospective	Zimbabwe	Zimbabwe	Women with/without KS and HIV/AIDS	41	ELISA	0	41	0	31.5 (1 -62 years)	85%	Serum, PBMC, vaginal, endocervical,	A5	X
[57]	Retrospective	Zimbabwe	Zimbabwe	AIDS patients	3571	ELISA	2131	1440	145	32.5 (0 -65 years)	18.2%	X	X	X
[103]	Retrospective	Zimbabwe	Zimbabwe	AIDS-KS patients	65	X	50	15	0	45 (20 -72 years)	X	Blood	A5, B	X
[29]	Prospective	Zimbabwe	Zimbabwe	AIDS-KS patients	65	X	50	15	0	42 (18 -66 years)	X	Blood	A5, B	X
[58]	Retrospective	Malawi	Malawi	Hospitalized patients and healthy volunteers	272	K8.1 ELISA	109	143	20	32.5 (1 -64 years)	67%	X	X	X
[104]	Retrospective	Malawi	Malawi	AIDS-KS patients	5	X	3	2	0	19.5 (7 -32 years)	X	Mouth rinse and blood	A2, A5, B	X
[105]	Prospective	UK	Malawi	AIDS-KS	22	IFA	11	11	0	48.5 (2 -95 years)	X	Mouth rinse and blood	A, B	X

Note: X = no data exist on the parameter, N/M= No mention, PBMC = Peripheral Blood Mononuclear Cells, LANA= Latency-associated nuclear antigen, ELISA = Enzyme-linked Immunosorbent Assay, IFA = Immunofluorescence Assay, WB= Western Blot, KS = Kaposi's Sarcoma, FI = Febrile Infant. N* = first report of subtype N using ORF75 gene. There are 9 countries in Southern Africa, of which 6 countries had published data meeting the search criteria and date of search.

Table S2: Characteristics of studies on Central African countries included in the analysis

Reference	Study type	Country of study	Country of participants	Study population	Sample size	Type of assay for sero-prevalence study	No of males	No of females	No of children	Average Age(Age range of participants)	Percentage sero-prevalence	Source of DNA/Antibodies for serology/genotyping	K1 genotypes	K15 genotypes
[13]	Retrospective	Cameroon	Cameroon	HIV +ve and HIV –ve individuals from the rural setting	516	ELISA and IFA	N/M	N/M	N/M	N/M	61%	Plasma	X	X
[101]	Prospective	France	Cameroon	AIDS-KS patients	49	X	36	18	N/M	49 (20 -78 years)	X	KS biopsy	A5, B1, B2	M, P
[59]	Retrospective	Cameroon	Cameroon	Hospital setting	16	ELISA	N/M	N/M	N/M	35.5 (31 – 40 years)	42.8%	Plasma	X	X
[30]	Prospective	Italy	Cameroon	AIDS-KS patients	3	X	3	0	0	45.5 (21 -70 years)	X	KS biopsy	A5	M, P
[8]	Prospective	Cameroon	Cameroon	HIV +ve, -ve, KS patients	1177	ELISA	N/M	N/M	N/M	34.5 (19 -50 years)	80%	Plasma	X	X
[39]	Retrospective	Cameroon	Cameroon	Rural setting of all aged groups	2063	LANA IFA	1,096	967	0	44.5 (2 -87 years)	37.2%	KS biopsy	A1-4, A5, B1	P
[60]	Prospective	Cameroon	Cameroon	Population based	293	ELISA	129	164	0	32.5 (1 -64 years)	50.5%	X	X	X
[61]	Prospective	USA	Cameroon	AIDS-KS patients	38	ELISA	38	0	0	38 (31 -45 years)	61%	X	X	X
[61]	Prospective	USA	Cameroon	General population	3123	ELISA and IFA	N/M	N/M	N/M	N/M	29.6%	X	X	X
[62]	Retrospective	Cameroon	Cameroon	Pregnant women and prostitutes	274	IMP	N/M	274	N/M	12 (11 -13 years)	38-57%	Serum	X	X
[18]	Retrospective	France	Cameroon	Hospital based-mother-child pair survey	258<br>32	IFA<br>IFA	X<br>X	X<br>32	258<br>X	N/M	27.5%<br>47%	Plasma<br>Plasma	X<br>X	X<br>X

[63]	Retrospective	Cameroon	Cameroon	Serosurvey-Hospital based	292	IFA	129	65	98	22.5 (5 -40 years)	39.8-61.8%	Serum	X	X
[12]	Prospective	Cameroon	Cameroon	Survey	608	ELISA	134	165	309	44.5 (1 -88 years)	59.9	plasma	X	X
[64]	Retrospective	Cameroon	Paris	Pregnant women	567	ELISA	0	567	0	30 (25 – 30 years)	45.6%	Plasma	X	X
[101]	Prospective	France	Central Africa Republic	AIDS-KS + HIV +ve MCD patients	12	X	6	6	0	49 (20 – 78 years)	X	KS biopsy + lymph nodes	A5, B1, B2, C	M, P
[37]	Retrospective	France	Central Africa Republic	AIDS-KS patients	3	IFA and IMP	2	1	0	22.5 (20 – 25 years)	100%	Blood	A5	X
[65]	Retrospective	Central Africa Republic	Central Africa Republic	AIDS-KS patients	36	Lytic and LANA IFA	15	21	0	31.5 (8 – 50 years)	74%<br>71%	Plasma	X	X
[26]	Prospective	England	Congo	AIDS-KS patients	23	ELISA	N/M	N/M	N/M	45 (15 – 75 years)	17.4%	X	X	X
[101]	Prospective	France	Congo	MCD-HIV +ve patients	1	X	1	0	0	49 (20 – 78 years)	X	Tonsil	B1	M
[66]	Prospective	Gabon	Gabon	Hospital based	344	ELISA	0	344	N/M	31.5 (14 – 49 years)	31%	X	X	X
[41]	Retrospective	USA	Democratic Republic of Congo (Zaire)	Community based Case-control	321	ELISA and IFA	155	166	N/M	N/M	82%	Serum/Plasma	X	X
[100]	Restrospective	USA	Democratic Republic of Congo (Zaire)	Endemic KS patients	1	X	1	0	0	N/M	X	KS biopsy	B	X
[38]	Retrospective	USA	Democratic Republic of Congo (Zaire)	Endemic KS patients	1	X	1	0	0	N/M	X	KS biopsy	X	P

Note: X = no data exist on the parameter, N/M= No mention, LANA= Latency-associated nuclear antigen, ELISA = Enzyme-linked Immunosorbent Assay, IFA = Immunofluorescence Assay, IMP = Immunoperoxidase , KS = Kaposi's Sarcoma, MCD = Multicentric Castleman Disease. There are 9 countries in Central Africa, of which 5 countries had published data meeting the search criteria and date of search.

Table S3: Characteristics of studies on West African countries included in the analysis

Reference	Study type	Country of study	Country of participants	Study population	Sample size	Type of assay for sero-prevalence study	No of males	No of females	No of children	Average Age (Age range of participants)	Percentage sero-prevalence	Source of DNA/Antibodies for serology/genotyping	K1 genotypes	K15 genotypes
[68]	Prospective	Nigeria	Nigeria	Hospital based	180	ELISA	60	120	0	39.5 (15 -64 years)	87%	Plasma	X	X
[69]	Comparative	USA	Nigeria	General population	1085	K8.1 and LANA ELISA	0	1085	N/M	39.5 (25 -54 years)	46.02%	Plasma	X	X
[70]	Cross sectional	Nigeria	Nigeria	AIDS patients with KS KS patients without HIV	71	ELISA	23	48	0	29.5 (19 -40 years)	62%	Plasma	X	X
[71]	Retrospective	Nigeria	Nigeria	Population based	1880	ELISA	718	1162	0	25 (0 -50 years)	23.5%	X	X	X
[72]	Prospective	Burkina Faso	Burkina Faso	Pregnant women (HIV +ve, -ve and AIDS patients)	429	ELISA IFA	0	0	429	31 (18 – 44 years)	11.4%	Serum	X	X
[73]	Prospective	Burkina Faso	Burkina Faso	Hospital based	683	ELISA	191	492	0	25.3 (16 -45 years)	12%	X	X	X
[105]	Retrospective	UK	Gambia	AIDS-KS	9	X	8	1	0	48.5 (2 -95 years)	X	KS Biopsy	B	X
[74]	Retrospective	Ghana	Gambia	Hospital based	N/M	ELISA and IFA	N/M	N/M	N/M	N/M	73-83%	Serum	X	X
[44]	Retrospective	USA	Ghana	Rural and urban setting	62	ELISA	47	15	0	42.5 (17 -68 years)	41.9%	X	X	X
[75]	Retrospective	Ghana	Ghana	Hospital based	410	ELISA	365	45	0	N/M	33.5-43.0	Serum	X	X
[76]	Retrospective	Ghana	Ghana	Hospital based	3275	ELISA	2573	702	0	41.5 (18 – 65 years)	65.6%	X	X	X

[26]	Prospective	UK	Ghana	AIDS-KS patients	56	ELISA	N/M	N/M	N/M	45 (15 – 75 years)	23.2%	X	X	X
[57]	Retrospective	France	Togo	HIV + MCD patients	1	X	1	0	0	N/M	X	Spleen	B	M
[101]	Retrospective	France	Senegal	AIDS-KS and IKS	2	X	2	0	0	49 (20 -78 years)	X	KS biopsy	B2, C	P, M
[77]	Retrospective	Senegal	Senegal	HIV + pregnant women	407	IFA	0	407	0	29.24 (N/M)	14.3%	Serum	X	X
[101]	Retrospective	France	Mauritania	MCD	1	X	1	0	0	N/M	X	Lymph node	A	N/M

Note: X = no data exist on the parameter, N/M= No mention, LANA= Latency-associated nuclear antigen, ELISA = Enzyme-linked Immunosorbent Assay, IFA = Immunofluorescence Assay, KS = Kaposi's Sarcoma, MCD = Multicentric Castleman Disease, IKS = Iatrogenic Kaposi's Sarcoma. There are 15 countries in Western African countries, of which 7 countries have published data meeting the search criteria and date of search.

Table S4: Characteristics of studies on East African countries included in the analysis

Reference	Study type	Country of study	Country of participants	Study population	Sample size	Type of assay for sero-prevalence study	No of males	No of females	No of children	Average Age (Age range of participants)	Percentage sero-prevalence	Source of DNA/Antibodies for serology/genotyping	K1 genotypes	K15 genotypes
[100]	Retrospective	Tanzania	Tanzania	Hospital based	248	ELISA	97	151	0	25 /(0 -50 years)	33.8%	X	X	X
[78]	Prospective	Sweden	Tanzania	Hospital based	78	ELISA	24	54	0	38 (No range mentioned)	69%	Serum	X	X
[79]	Prospective	Tanzania	Tanzania	Rural based	798	ELISA	357	379	62	N/M	54-93%	Serum	X	X
[74]	Prospective	Sweden	Tanzania	Hospital based	184	ELISA and IFA	157	27	0	38 (No range mentioned)	89%	Serum	X	X
[26]	Prospective	UK	Tanzania	Hospital based	23	ELISA	N/M	N/M	N/M	41.5 (15 -75 years)	17.4%	X	X	X
[38]	Retrospective	USA	Tanzania	AIDS-KS patients	2	X	2	0	0	N/M	X	KS Biopsy	A5, B	P
[80]	Retrospective	Kenya	Kenya	Hospital based	633	ELISA	0	633	0	26 (22 -31 years)	44.1%	X	X	X
[81]	Prospective	USA	Kenya	Female sex workers	354	ELISA	0	354	0	41 (3 -79 years)	4.5%	X	X	X
[61]	Prospective	USA	Kenya	KS patients	39	ELISA	39	0	0	38 (31 – 45 years)	29.6%	X	X	X
[82]	Retrospective	Kenya	Kenya	Population based	1061	ELISA	1061	0	0	35 (30 -40 years)	43%	X	X	X
[83]	Prospective	Kenya	Kenya	Population based female prostitute	174	ELISA	0	174	0	32 (18 -46 years)	26.0%	X	X	X
[26]	Prospective	UK	Kenya	Hospital based	23	ELISA	N/M	N/M	N/M	45 (15 -75 years)	17.4%	X	X	X
[30]	Prospective	Kenya	Kenya	AIDS-KS	68	X	5	1	62	N/M	X	KS biopsy	A5, B1, C, F	P
[84]	Prospective	Kenya	Kenya	Hospital based	228	LANA	0	228	0	N/M	63.5%	X	X	X
[100]	Retrospective	Uganda	Uganda	Hospital based	3196	ELISA	N/M	N/M	2404	N/M	42.4-70.6%	X	X	X

[85]	Retrospective	UK	Uganda	Hospital based	215	ELISA, IFA and WB	76	139	35	1 day – 24 years	33-52%	Serum	X	X
[62]	Retrospective	Uganda	Uganda	Hospital based	118	IFA	0	15	103	25 (11 -39 years)	88%	Serum	X	X
[86]	Retrospective	USA	Uganda	Random section-population based	523	ELISA and IFA	239	283	0	22 (15 -29 years)	38.5%	Serum	X	X
[106]	Prospective	Uganda	Uganda	AIDS-KS	4	X	3	1	0	N/M	X	KS biopsy	A, B	X
[107]	Retrospective	Uganda	Uganda	KS patients	56	X	N/M	N/M	N/M	N/M	X	Blood	A5, B, C, F	X
[87]	Prospective	Uganda	Uganda	Cross-sectional; mother and child study	2391	ELISA	0	1164	1227	22 (14 -30 years)	15-69%	Serum	X	X
[88]	Retrospective	UK	Uganda	Endemic KS patients and blood donors	30<br>118	ELISA, IFA and WB	25<br>98	5<br>18	N/M<br>N/M	N/M	N/M<br>74%	KS biopsy<br>Plasma	A5, B, C	M, P
[30]	Prospective	Italy	Uganda	AIDS-KS and Endemic KS	14	X	13	1	0	45.5 (21 -70 years)	X	KS biopsy	A5, B1, B3, C	P
[38]	Retrospective	USA	Uganda	AIDS-KS patients	2	X	2	0	0	N/M	X	KS biopsy	B	P
[60]	Prospective	Uganda	Uganda	Hospital based	315	ELISA	42	273	0	32.5 (1 -64 years)	44.8%	X	X	X
[89]	Retrospective	Uganda	Uganda	Population based	2681	ELISA	1448	1233	0	29.5 (19 -40 years)	56.2%	X	X	X
[90]	Retrospective	Uganda	Uganda	Population based	1915	ELISA	0	1915	0	39.5 (14 -35 years)	32%	X	X	X
[50]	Retrospective	Uganda	Uganda	Population based	427	ELISA	N/M	N/M	427	5.2 (1.5 – 8.9 years)	9.0%	X	X	X
[44]	Retrospective	USA	Uganda	Hospital based	62	ELISA	44	18	0	42.5 (17 -68 years)	45.7%	X	X	X
[91]	Retrospective	Uganda	Uganda	Survey	2681	ELISA	N/M	N/M	N/M	37 (15 -59 years)	55.4%	X	X	X

[92]	Retrospective	Uganda	Uganda	Hospital based	461	ELISA	0	228	233	30 (15 -45 years)	43%	X	X	X
[108]	Retrospective	Uganda	Uganda	Hospital based	600	ELISA	0	600	0	11.5 (7 -16 years)	N/M	Saliva and buffy coat	A5, B, C	X
[105]	Retrospective	UK	Uganda	Hospital based	7	X	N/M	N/M	N/M	48.5 (2 -95 years)	X	PBMCs and biopsy	A5, B, C	X
[67]	Retrospective	USA	Uganda	Hospital based	3	X	N/M	N/M	N/M	N/M	X	Biopsy	A, B	X
[26]	Prospective	UK	Uganda	Hospital based	23	ELISA	N/M	N/M	N/M	45 (15 -75 years)	38.7%	X	X	X
[93]	Retrospective	Ethiopia	Ethiopia	Hospital based	202	ELISA ORF73	N/M	N/M	N/M	N/M	39.1%	Serum	X	X
[94]	Retrospective	Ethiopia	Ethiopia	Hospital based	400	ELISA ORF7	0	400	0	N/M	53%	X	X	X
[36]	Retrospective	Eritrea	Eritrea	Hospital based	411	IFA	0	227	184	22 (2 -42 years)	2%	Serum	X	X

Note: X = no data exist on the parameter, N/M= No mention, PBMC = Peripheral Blood Mononuclear Cells, LANA= Latency-associated nuclear antigen, ELISA = Enzyme-linked Immunosorbent Assay, IFA = Immunofluorescence Assay, WB= Western Blot, KS = Kaposi's Sarcoma. There are 12 countries in East Africa, of which 5 countries have published data meeting the search criteria and date of search.

Table S5: Characteristics of studies on North African countries included in the analysis

Reference	Study type	Country of study	Country of participants	Study population	Sample size	Type of assay for sero-prevalence study	No of males	No of females	No of children	Average Age (Age range of participants)	Percentage sero-prevalence	Source of DNA/Antibodies for serology/genotyping	K1 genotypes	K15 genotypes
[101]	Retrospective	France	Algeria	HIV+ MCD	1	X	1	0	0	49 (20 -78 years)	X	Spleen	C	P
[17]	Prospective	Egypt	Egypt	Hospital based	246	ELISA and IFA	126	0	196	10.5 (1 -20 years)	42.4% 7-45%	X	X	X
[95]	Prospective	Egypt	Egypt	Hospital based	86	IFA	0	0	86	36 (12 – 46 months)	41.9%	X	X	X
[85]	Retrospective	Egypt	Egypt	Random selection	965	ELISA	N/M	N/M	N/M	N/M	24.2%	X	X	X

[62]	Prospective	Egypt	Egypt	Population based	236	ELISA and IFA	120	116	0	32.5 (1 -64 years)	42.4% 42-43%	X	X	X
[96]	Prospective	Tunisia	Tunisia	Hospital based Pregnant women	553	ELISA	0	553	0	25 (20 -30 years)	13.8%	X	X	X
[97]	Prospective	Tunisia	Tunisia	Hospital based	60	ELISA	26	34	0	25 (20 -30 years)	17%	X	X	X
[98]	Prospective	Morocco	Morocco	KS patients	216	ELISA	N/M	N/M	N/M	N/M	28.7%	X	X	X
[35]	Prospective	Morocco	Morocco	KS patients	26	ELISA	N/M	N/M	N/M	61.5 (40 – 83 years)	92%	X	X	X
[109]	Prospective	Morocco	Morocco	AIDS-KS	28	X	20	8	0	51.5 (23 - 80 years)	X	Biopsy	A, B, C2, C3, C7 C''	M, P

Note: X = no data exist on the parameter, N/M= No mention, ELISA = Enzyme-linked Immunosorbent Assay, IFA = Immunofluorescence Assay, KS = Kaposi's Sarcoma, MCD = Multicentric Castleman Disease. There were 7 countries in Northern Africa, of which 4 countries had published data meeting the search criteria and date of search.
